# Delta brush variant: A novel ictal EEG pattern in anti‐NMDAR encephalitis

**DOI:** 10.1002/epi4.12423

**Published:** 2020-08-12

**Authors:** Qi Huang, Yuhan Liao, Meigang Ma, Yuan Wu

**Affiliations:** ^1^ Department of Neurology First Affiliated Hospital Guangxi Medical University Nanning China

**Keywords:** anti‐NMDAR encephalitis, delta brush, electroencephalogram pattern, seizure

## Abstract

Seizure is one of the main symptoms of anti‐NMDAR encephalitis, but data of ictal electroencephalogram (EEG) patterns remain limited. In this study, we aimed to introduce a unique ictal pattern. This delta brush variant (DBV) was characterized as generalized delta rhythm with fast spike activity riding on it. We retrospectively evaluated the ictal pattern from six patients with anti‐NMDAR encephalitis, and patients were grouped based on the presence of DBV. DBV was found in two patients who were in the florid phase of the disease: (a) A 17‐year‐old girl experienced rhythmical jerking of bilateral limbs. Corresponding EEG patterns showed generalized DBV. Seizure terminated after intravenous injection of midazolam, but oral‐facial dyskinesia reappeared; and (b) a 24‐year‐old man suffered stiffening of the right limbs and oral‐facial dyskinesia. The EEG pattern showed frontal DBV with left prominence. Seizure was controlled, but oral‐facial dyskinesia remained after intravenous injection of midazolam. Compared with patients without DBV, patients in this group were more likely to have prolonged excessive delta brush (100% vs 25%) and hyperpyrexia (39.7℃ vs 38.2℃). Duration in ICU (36 days vs 18 days) and hospital (52 days vs 36 days) was relatively longer in DBV group, and no significant difference was found in terms of the mRS score (1 vs 0.5) and seizure relapse rate (0% vs 25%) during 3‐month follow‐up. DBV is a peculiar pattern in anti‐NMDAR encephalitis. An EEG‐based monitoring should be considered to avoid misleading this ictal EEG pattern to the electromyographic artifact.

**Key Points**

- Delta brush variance is a novel ictal electroencephalogram pattern in anti‐NMDAR encephalitis.
- Delta brush variance is characterized as generalized delta rhythm with fast spike activity riding on it.
- Delta brush variance is derived from extreme delta brush in the florid phase of the disease.
- Cortex‐subcortical area interaction may contribute to the evolution between delta brush variance and extreme delta brush.

## 1 INTRODUCTION

Anti‐N‐methyl‐D‐aspartate receptor (NMDAR) encephalitis is the most common form of autoimmune encephalitis due to specific antibody crosslinking the NMDAR, causing receptor capping and internalization.^1^ The major symptoms of this disease include abnormal behavior, speech dysfunction, seizure, movement disorder, decreased consciousness level, and autonomic dysfunction.^2^ Diagnosis can be made based on the presence of proper symptoms and IgG anti‐GluN1 antibodies in cerebrospinal fluid (CSF).^3^ Extreme delta brush (EDB) and generalized rhythmic delta activity (RDA) are two frequent and prominent EEG patterns of anti‐NMDAR encephalitis.^4^ Although not specific, occasional neuroimages described related lesions in hippocampus, basal ganglia, and white matter.^2^ More than 80% patients with anti‐NMDAR encephalitis have good outcome after receiving standardized immunotherapy.^5^

Seizure is common with prevalence of 70%‐90% in all anti‐NMDAR cases.^6^, ^7^ Hitherto, studies on electroencephalogram (EEG) of anti‐NMDAR encephalitis showed that the most common epileptic discharge is regional sharp wave (71.6%), followed by periodic lateralized epileptiform discharge (19.4%), and generalized periodic epileptiform discharge(9.0%). Only 8% of the total cases were reported to have ictal patterns; among which, regional rhythmic alpha activity showed significant correlation with seizure occurrence in anti‐NMDAR encephalitis.^8^, ^9^ EEG plays a crucial role in distinguishing seizure and paroxysmal non‐epileptic events; however, the data of related ictal patterns remain limited.

Extreme delta brush is a unique EEG pattern in anti‐NMDA receptor encephalitis and is characterized as a RDA at 1‐3 Hz with superimposed bursts of rhythmic 20‐30 Hz beta frequency activity.^10^ This pattern was once considered to be an ictal pattern, because the patients were more likely to undergo prolonged coma.^11^ However, given that rare cases with EDB respond to sedative drugs electrographically, the clinical nature of this pattern remains controversial. In the present study, we introduced a unique ictal pattern evolving from EDB and discussed its significance in anti‐NMDAR encephalitis.

## 2 SUBJECTS AND METHODS

### 2.1 Definition

1‐Ictal EEG patterns: based on the American Clinical Neurophysiology Society Research Criteria for Nonconvulsive Seizure,^12^ an ictal pattern was characterized as (a) an unequivocal change in rhythmic pattern, lasting for more than 10 seconds, (b) weaning after intravenous injection of diazepam or midazolam, and (c) with or without clear clinical ictal symptoms.

2‐Delta brush variant (DBV): a pattern that satisfied (a) the criteria of ictal EEG patterns, and (b) the key feature of EDB, namely a complex delta activity with superimposing fast activity or intermixed spikes.

### 2.2 Patients inclusion and grouping

Data were collected from the First Affiliated Hospital in Guangxi Medical University. This study involved human participants and was reviewed and approved by the hospital ethics committee. Patients from January 2012 to December 2019 who (a) had acute onset of neurological or psychiatric symptoms, (b) had confirmed serum and CSF NMDAR antibodies evaluated by specific staining against NMDAR isolated from rats’ hippocampus and cerebellum, and positive cell‐based assay with HEK293 cells transfected with NR1, and (c) underwent at least 4‐hour continuous EEG monitoring and presented ictal EEG patterns were included. The exclusion criteria included (a) patients diagnosed with epilepsy, cerebral infarction, cerebral trauma, cerebral tumor, and other nervous system disease prior to the onset of encephalitis, (b) patients with evidence of infectious encephalitis, for example, viral, bacteria, Mycobacterium tuberculosis, or fungi. Patients were grouped according to the presence of DBV.

Clinical data were extracted retrospectively. Information regarding age, gender, semiology, neuroimaging findings, CSF findings, EEG features, immunotherapy, drug responses, and outcome at 3 months was collected. The continuous EEG recordings were reviewed independently by two internists (HQ and MM) who passed the National Examination of EEG Technology of China. The following variables related to EEG characteristics were collected: preictal and ictal electrographic patterns, and corresponding semiology.

### 2.3 Statistical analysis

Data were statistically analyzed using Stata 13. Between‐group difference of categorical and quantitative variables was compared using Fisher's exact test and Wilcoxon rank‐sum test respectively.

## 3 RESULTS

The initial dataset of anti‐NMDAR encephalitis contained 72 cases. Ictal patterns presented in 6 (8.3%) cases including four females and two males with mean age 21.3 years (range 17–30 years). DBV was performed in the following two cases.

### 3.1 Case 1

**FIGURE 1:**
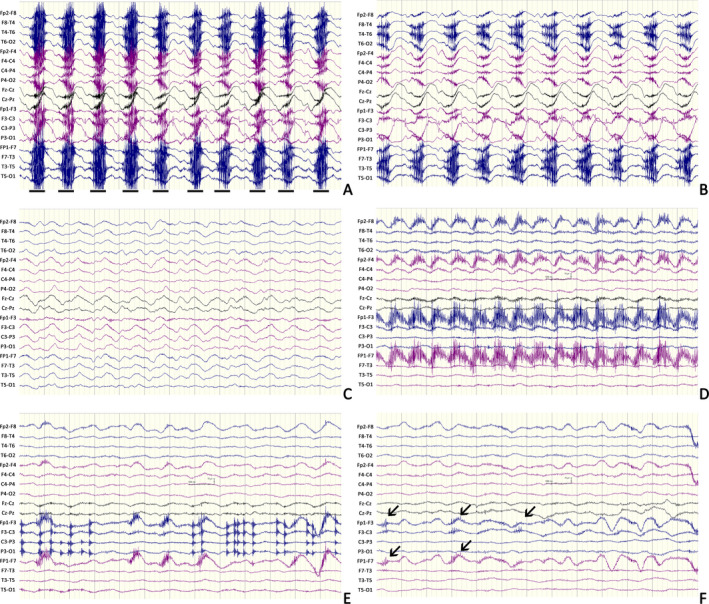
Representative EEG patterns of patient with delta brush variance. Patient #1 (A‐C): A, Generalized rhythmic delta activity (RDA) with periodic fast spike activity riding on it; clonus was locked to each fast spike epochs (black bar). B, 30 s later after intravenous injection of midazolam at a dose of 0.2 mg/kg; seizure alleviated but oral‐facial dyskinesia reappeared. C, Typical extreme delta brush recovered 10 min later after injection; no seizure but the patient showed prolonged oral‐facial dyskinesia and choreic movement. Panel A & B recorded pop artefacts on C3. Patient #2 (D‐F): D, Generalized 1.2 Hz delta rhythm with left frontal prominent fast spike activity; the patient experienced focal tonic seizure of right limbs and oral‐facial dyskinesia. E, 30 s later after intravenous injection of midazolam at a dose of 0.2mg/kg; seizure alleviated but oral‐facial dyskinesia remained. F, 10 min later after injection, EEG showed an extreme delta brush with paroxysm low‐voltage spike activity (black arrow); no seizure but the patient showed prolonged oral‐facial dyskinesia. Panel E recorded muscular artefacts on C3, P3, and O1. Recording parameters: sensitivity 10 µV/mm, high‐pass filter 0.5 Hz, low‐pass filter 70 Hz. Channels setting (from top to bottom): Fp2‐F8, F8‐T4, T4‐T6, T6‐O2, Fp2‐F4, F4‐C4, C4‐P4, P4‐O2, Fz‐Cz, Cz‐Pz, Fp1‐F3, F3‐C3, C3‐P3, P3‐O1, Fp1‐F7, F7‐T3, T3‐T5, T5‐O1

A 17‐year‐old girl experiencing progressive psychosis, agitation, and oral‐facial dyskinesia for 4 days was admitted. The initial EEG results showed typical generalized EDB. Diffuse low‐density lesion involving the white matter of the bilateral temporal and occipital lobes was noted by computerized tomography (CT) scan. She was diagnosed as anti‐NMDAR encephalitis after NMDAR antibody was detected in her serum and CSF. Glucocorticoid with a dose of 500 mg/day was given but symptoms remained. Over the next 3 weeks, the patient developed hyperpyrexia, severe pneumonia, and pyothorax. On day 23, she began to have rhythmical jerking of bilateral limbs with cessation of choreic movement and oral‐facial dyskinesia. Corresponding EEG patterns showed generalized synchronous delta rhythm with fast spike activity riding on it (Figure 1A‐C). Seizure terminated after intravenous injection of midazolam at a dose of 0.2 mg/kg, but oral‐facial dyskinesia reappeared. Midazolam at a dose of 0.2 mg/kg/h was continuously infused and terminated 2 days later. Subsequent immunotherapy (plasma exchange, intravenous immunoglobulin, and cyclophosphamide) was given routinely, and she recovered with a mRS score of 1 after 3 months. No seizure was reported after withdrawing AEDs (Table S1).

### 3.2 Case 2

A 24‐year‐old man suffering psychosis and recurrent focal tonic seizures of right limbs for 7 days was admitted. Prolonged fever, right hypesthesia, and stiffness of right face were also reported. The initial EEG results presented generalized RDA. Magnetic resonance imaging (MRI) scan showed nothing remarkable. A NMDAR antibody with titer of 1:32 was detected in his CSF. Glucocorticoid with a dose of 1000 mg/day was given, but the patient developed aggravating impaired awareness and began to have oral‐facial dyskinesia. On day 6 in the hospital, focal status epilepticus with stiffening of the right arm and leg was recorded. The EEG pattern showed RDA with frontal fast spike activity, most prominent over the left region. Seizure was controlled but oral‐facial dyskinesia remained after intravenous injection of midazolam at a dose of 0.2 mg/kg (Figure 1D‐F). The patient successively received intravenous immunoglobulin and AEDs. After 3 months, the patient had a mRS score of 1. The patient was treated with oxcarbazepine at a dose of 600 mg/day, and no seizure was reported (Table S1).

### 3.3 Association of DBV with clinical features

About 75% seizure occurrences in non‐DBV group were non‐convulsive, and this percentage was higher than that in the DBV group. To detect factors causing DBV, we compared the preictal clinical data between groups. A relatively higher rate of prevalence of EDB (100% vs 25%) and lower GCS score (3.75 vs 6.62) was presented in the DBV group. And malignant hyperthermia was more likely to be accompanied with DBV (39.7℃ vs 38.2℃). Furthermore, a total of 3 (75%) patients in the non‐DBV group were sensitive to intravenous diazepam, showing better response than those in the DBV group (Table S2).

Compared with the non‐DBV group, patients with DBV had longer time in ICU (36 days vs 18 days) and hospital (52 days vs 36 days). We used mRS score to assess the recovery of encephalitis on the 3rd month, and no significance was found between groups (1 vs 0.5). All patients received two or more kinds of antiepileptic drugs. One female (Patient 4, see Table S1) in the non‐DBV group relapsed during oxcarbazepine reduction. Difference of seizure recurrence was insignificant during the 3‐month follow‐up (Table S2).

## 4 DISCUSSION

In general, we introduced a novel ictal EEG pattern in anti‐NMDAR encephalitis. The presented DBV was characterized as generalized delta activity with superimposing fast spike activity. This pattern showed predominance in the florid phase of the disease. Patients with continuous EDB and prolonged hyperpyrexia tend to develop DBV. Patients with DBV were likely to spend more time in ICU and hospital, but the long‐term outcome and seizure relapse rate were similar to those in patients without it.

To our knowledge, this pattern has not been described in anti‐NMDAR encephalitis. As movement disorder is more common in anti‐NMDAR encephalitis, ictal patterns should be carefully compared with electromyographic (EMG) signal to avoid misleading results. In general, the potentials generated in the muscles are of shorter duration than those generated in the brain. The frequency components are usually beyond 30‐50 Hz.^13^ Although DBV seems similar to an EMG artifact at first look, several considerations support the epileptic nature of this pattern. First, the proposed fast component in DBV is a generalized pattern, while EMG always shows frontal or temporal predominance. Second, the burst of fast component is periodic, while EMG is always arrhythmic. Third, the fast component is locked to unequivocal seizures, while EMG is not. Finally, the movement disorder‐induced artifact rarely responds to sedative, while DBV weans after intravenous injecting of midazolam.

EDB is one of the key features in anti‐NMDAR encephalitis and regarded as a potential marker for the disease with a prevalence of 30% in total patients.^4^ Thus far, the ictal nature of EDB remains unclear partly because of the semiological mismatching. Previous studies revealed that EDB indicates forthcoming electroclinical seizures,^11^ suggesting that it is an preictal state of status epilepticus in NMDAR encephalitis. By contrast, the inertness to benzodiazepines and AEDs shows that EDB itself is prone to an interictal pattern, which is related to the florid phase of encephalopathy.^10^ We named this pattern DBV because of its resemblance to the well‐known EDB. Our DBV typically appears as a combination of clusters of fast spike activity and delta wave. Compared with EDB, the fast component of DBV shows a higher frequency and amplitude. Furthermore, the fast spike activity tends to alleviate after midazolam injection, leading DBV to be more epileptic than EDB.^14^ Moreover, the ictal nature of DBV was also supported by EEG terminology criterion proposed by American Clinical Neurophysiology Society.^15^ According to that criterion, DBV can be standardized as rhythmic delta activity superimposing spikes (RDA + S). It is noted that additional spikes which renders the delta activity make the EEG pattern more ictal‐appearing than the usual term without the plus.

In fact, determining an ictal discharge is one of the most challenging issue for anti‐NMDAR encephalitis. Most paroxysmal attacks of psychomotor agitation need to be recorded by EEG to avoid mistakenly diagnosing as seizures, and most of paroxysmal rhythmic activities in anti‐NMDAR encephalitis are non‐epileptic.^16^ As a result, recognition of an ictal pattern is important for clinical practice. Several recent studies have demonstrated that persistent rhythmic delta and alpha‐like activities are predictors of ictal events,^17^, ^18^ and our study extends this area by introducing DBV. Compared with these patterns which corresponds to non‐convulsive status epilepticus (NCSE), DBV is more likely to be accompanied with motor seizures. Moreover, both cases of DBV were recorded during the florid phase, which was quite different to those are common at the initial stage of anti‐NMDAR encephalitis. Finally, previous ictal patterns show close relationship to teratoma,^17^, ^19^ but no teratoma was detected in the two cases with DBV.

**FIGURE 2:**
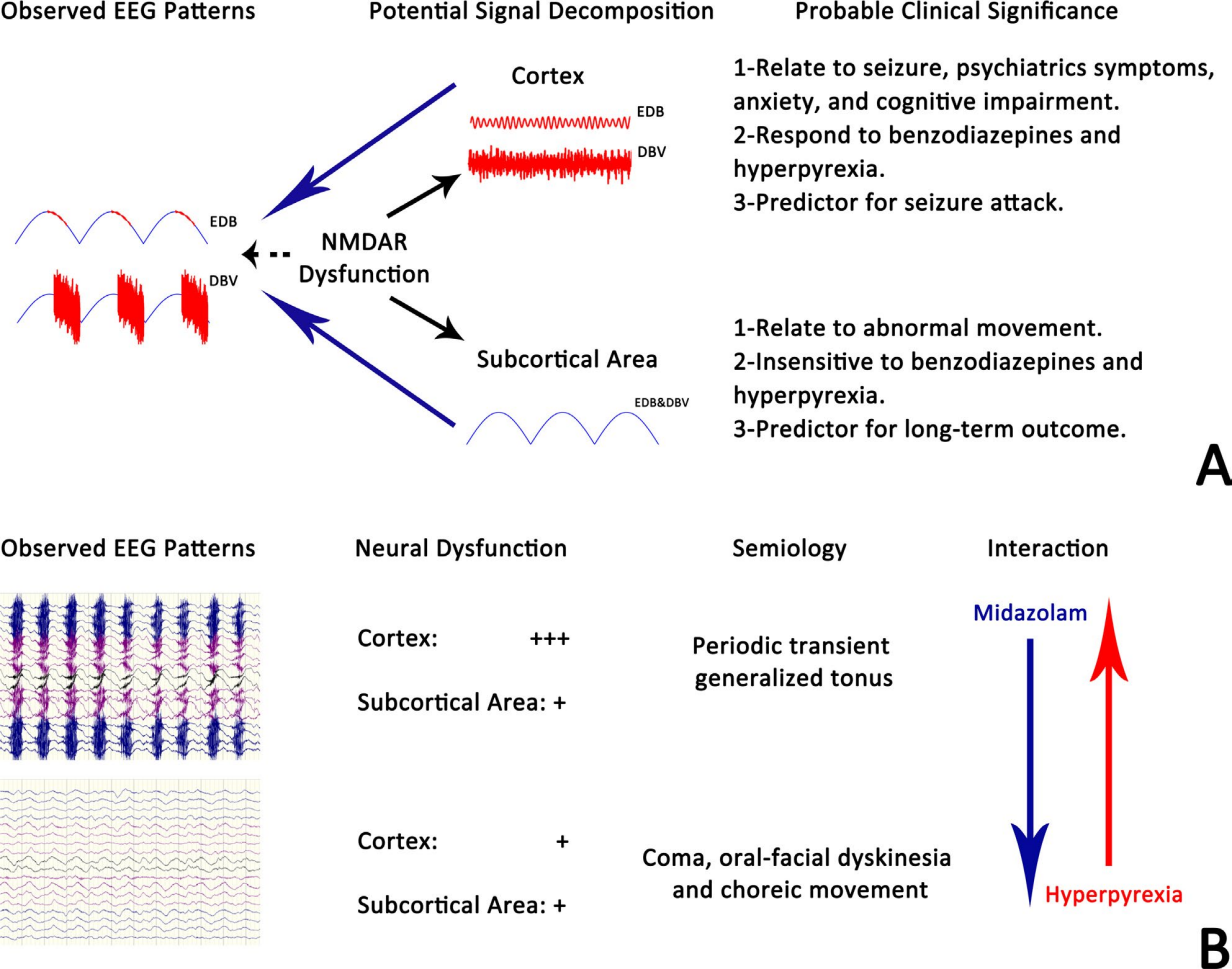
Cortical and subcortical dysfunction in anti‐NMDAR encephalitis. A, Diagram detailing the physiopathological hypothesis of EDB & DBV complexes. B, Diagram detailing the temporal evolution of EEG abnormalities and semiology observed in seizure. EDB, excessive delta brush; DBV, delta brush variant

The semblable morphology of DBV and EDB indicated that dysfunction of NMDAR might lead to various EEG phenotypes. In a retrospective study, Jeannin^4^ grouped the EEG abnormities in anti‐NMDAR encephalitis into excessive beta activity (EBA), generalized RDA, and EDB. They found that generalized RDA was associated with abnormal movement, while EBA and EDB which contain excessive fast activity were more likely to be accompanied with seizure, psychiatrics symptoms, anxiety, and cognitive impairment. Thus, they hypothesized that the presence of EBA and EDB was related to cortical dysfunction, and generalized RDA was presented when subcortical area involved. Our findings reinforced Jeannin's hypothesis by providing the evolution of the fast component during interictal‐ictal phase. Moreover, we also hypothesize that EDB is a superposition that contains signal from the cortex to form fast component (FC) and signal from subcortical area to form the delta component (DC). FC was sensitive to hyperpyrexia and benzodiazepines, and contributed to seizure, while DC showed an opposite feature. Alternation in cortical and subcortical involvement, which resulted in the composite changes in FC and DC as observed in scalp EEG, might be one of the primary causes of clinical evolution in anti‐NMDAR encephalitis (Figure 2).

Another finding of this study was that the presence of DBV had a similar mRS score and seizure relapse rate during a 3‐month follow‐up. Our observations, combined with those of other investigators, suggest that the relationship between EEG features and outcomes is debated.^20^ Our results supported that sedative is necessary to terminate DBV‐induced seizure, but the significance of immunotherapy cannot be ignored. One retrospective study which focused on the outcome of AEDs alone in controlling the seizure of patients with autoimmune encephalitis demonstrated that only 23.5% of patients became seizure‐free compared with 61.5% of patients with immunotherapy,^21^ indicating that the earlier and more intense immune treatment is undoubtedly more crucial for a better outcome. Moreover, the debate on the relationship between outcome and presence of DBV is still open due to the 3‐month follow‐up. Studies based on subtle clinical assessment and longer follow‐up are needed to draw the final conclusion.

The main purpose of the study is to introduce a unique ictal EEG pattern to avoid misdiagnosis. However, several limitations should be noted in the interpretation of DBV. First, the relatively small sample makes it difficult to determine the overall features of DBV. This pattern tends to be accompanied with continuous EDB and prolonged hyperpyrexia. The potential necessary combination of these two factors may explain why such a pattern was not reported previously. As DBV is recorded in the acute phase of anti‐NMDAR encephalitis when choreic movement is more frequent than seizure, more EEG‐based studies are needed to analyze the incidence and clinical nature of DBV. Second, only 8.3% (6/72) anti‐NMDAR encephalitis patients had recorded seizures in our study, which was relatively lower than other reports.^6^ This discrimination may partly result from the duration of continuous EEG monitoring. It is reported that comatose patients are more likely to have their first seizure recorded after >24 hours of monitoring.^22^ Thus, the presence of DBV remains unclear and needs verifying by extended continuous EEG monitoring. Third, based on the morphological and clinical relationship between DBV and EDB, we hypothesize that EDB is a kind of complex that reflects dysfunction from cortex and subcortical area, but the speculative nature of our observation still need further experiments to verify.

## CONFLICTS OF INTEREST

The authors declared no conflicts of interest with respect to the research, authorship, and publication of this article. We confirm that we have read the Journal's position on issues involved in ethical publication and affirm that this report is consistent with those guidelines.

## Supporting information

File S1

File S2
