# Supplementary material for: Delta brush variant: A novel ictal EEG pattern in anti‐NMDAR encephalitis
Source: Epilepsia Open. 2020 Aug 12;5(3):507–13. doi: 10.1002/epi4.12423 (PMC7469758; doi:10.1002/epi4.12423)
Supplement: Supplementary file 1 — File S1 [file EPI4-5-507-s001.docx]

| **Supplementary file 1 Detail of EEG patterns of included patients** | | | | | | | | | | | |
| --- | --- | --- | --- | --- | --- | --- | --- | --- | --- | --- | --- |
|  | | | | | | | | | | | |
|  | **Group** | **Gender** | **Age** | **Neuroimage** | **Immunotherapy** | **Preictal Characteristics** | |  | **Ictal Characteristics** | | |
|  |  |  |  |  |  | **Semiology** | **EEG pattern** |  | **Semiology** | **EEG pattern** | **Drug response** |
| **patient 1** | DBV | Female | 17 | CT:  Diffuse lesions involved white matter of the bilateral temporal and occipital lobes | Corticosteroid, Plasma exchange, Immunoglobulin | Coma, oral-facial dyskinesia and choreic movement | Generalized EDB |  | Generalized clonic seizure of bilateral limbs | Generalized RDA with superimposed fast spike activity | Seizure terminated after intravenous injection of midazolam, but oral-facial dyskinesia reappeared |
| **patient 2** | DBV | Male | 24 | MRI:  Negative | Corticosteroid, Plasma exchange, Immunoglobulin | Coma, and oral-facial dyskinesia | Generalized EDB, left predominance |  | Focal tonic seizure of right limbs; Oral-facial dyskinesia | Generalized RDA with frontal fast spike activity, most prominent over the left region | Seizure terminated after intravenous injection of midazolam, but oral-facial dyskinesia remained |
| **patient 3** | Non-DBV | Female | 30 | MRI:  Negative | Corticosteroid | Confusion | Generalized slowing at 6-7Hz |  | Mild facial twitching | Rhythmic alpha waves (10Hz) in right temporal area | **^*^**NCSE terminated after intravenous injection of diazepam |
| **patient 4** | Non-DBV | Female | 21 | MRI:  T2 hyperintensity in the bilateral hippocampus and posterior temporal lobe | Corticosteroid  Immunoglobulin | Psychosis and choreic movement | Generalized slowing at 6-7Hz, with episodic frontal 15-18Hz activity |  | Psychosis and choreic movement | Rhythmic sharp waves (3Hz) in right occipital area | **^*^**NCSE terminated after intravenous injection of diazepam |
| **patient 5** | Non-DBV | Male | 18 | CT:  Negative | Corticosteroid  Immunoglobulin | Psychosis, choreic movement, and hypoventilation | Generalized excessive beta activity |  | Focal to bilateral tonic–clonic seizure | Rhythmic theta waves (6.5Hz) in right temporal area → Generalized fast spike activity | Seizure terminated after intravenous injection of midazolam |
| **patient 6** | Non-DBV | Female | 18 | MRI:  Negative | Corticosteroid | Coma and oral-facial dyskinesia | Generalized EDB |  | Coma; alleviated oral-facial dyskinesia | Rhythmic alpha waves (10Hz) in parietal and occipital area | **^*^**NCSE terminated after intravenous injection of diazepam |

DBV: Delta Brush Variant; RDA: Rhythmic delta activity; NCSE: Nonconvulsive status epilepticus.

*: No motor characteristics was observed during the ictal phase in these patients. The diagnosis of NCSE was based on the electroencephalography changes (Beniczky S, et al. Epilepsia. 2013).
