# Supplementary material for: Delta brush variant: A novel ictal EEG pattern in anti‐NMDAR encephalitis
Source: Epilepsia Open. 2020 Aug 12;5(3):507–13. doi: 10.1002/epi4.12423 (PMC7469758; doi:10.1002/epi4.12423)
Supplement: Supplementary file 2 — File S2 [file EPI4-5-507-s002.docx]

| **Supplementary file 2 Clinical characteristics and outcomes between groups** | | | |
| --- | --- | --- | --- |
|  | | | |
|  | **DBV** | **Non-DBV** | **P value** |
| **Gender(male/female)** | 1/1 | 1/3 | 1.000 |
| **Age(mean±SD)** | 20.5±4.95 | 21.75±5.68 | 0.638 |
| **Initial symptom** |  |  |  |
| **Seizure (yes/no)** | 2/0 | 4/0 | - |
| **Psychiatric behave (yes/no)** | 2/0 | 3/1 | 1.000 |
| **Movement disorder (yes/no)** | 1/1 | 1/3 | 1.000 |
| **Neuroimage Finding(positive/negative)** | 1/1 | 1/3 | 1.000 |
| **CSF Antibody titer(mean±SD)^*^** | -1.8±0.35 | -1.5±0.41 | 0.453 |
| **Teratoma(yes/no)** | 0/2 | 1/3 | 1.000 |
| **Clinical features around ictal events** |  |  |  |
| **Glasgow Coma Scale score(mean±SD)** | 3.75±1.06 | 6.62±3.40 | 0.340 |
| **Temperature(mean±SD)** | 39.7±0.71 | 38.2±0.34 | 0.060 |
| **Presence of preictal EDB (Yes/No)** | 2/0 | 1/3 | 0.400 |
| **Seizure initial(generalized/focal)** | 1/1 | 0/4 | 0.333 |
| **Seizure type(motor/nonmotor)** | 2/0 | 1/3 | 0.400 |
| **Diazepam response(yes/no)** | 0/2 | 3/1 | 0.400 |
| **Immunotherapy** |  |  |  |
| **Starting time (days from onset)** | 7.0±3.5 | 6.0±2.2 | 0.481 |
| **Corticosteroids (yes/no)** | 2/0 | 4/0 | - |
| **Immunoglobulins (yes/no)** | 2/0 | 2/2 | 0.467 |
| **Plasma exchange (yes/no)** | 2/0 | 0/4 | 0.067 |
| **Anti-epileptic drug (single/combine)** | 0/2 | 0/4 | - |
| **Clinical outcomes** |  |  |  |
| **Duration in ICU(mean±SD)** | 36±4.2 | 18±10.4 | 0.064 |
| **Duration in hospital(mean±SD)** | 52±12.7 | 36±10.8 | 0.165 |
| **mRS score on the third month(mean±SD)** | 1±0.0 | 0.5±0.5 | 0.264 |
| **Seizure relapse (yes/no)** | 0/2 | 3/1 | 1.000 |

*: Logarithmic converting before statistical analysis
